# Occurrence of *Coxiella burnetii, Ehrlichia canis, Rickettsia* species and *Anaplasma phagocytophilum*-like bacterium in ticks collected from dogs and cats in South Africa

**DOI:** 10.4102/jsava.v88i0.1390

**Published:** 2017-05-19

**Authors:** Khethiwe Mtshali, Ryo Nakao, Chihiro Sugimoto, Oriel Thekisoe

**Affiliations:** 1 Department of Biomedical Sciences, Veterinary Technology Program, Tshwane University of Technology, South Africa; 2 Department of Zoology and Entomology, University of the Free State, South Africa; 3 Research Center for Zoonosis Control, Hokkaido University, Japan; 4 Unit for Environmental Sciences and Management, North-West University, South Africa

## Abstract

Ticks are major vectors of arthropod-borne infections and transmit a wide variety of zoonotic pathogens. This study was conducted mainly to determine the occurrence of canine tick-borne bacterial and rickettsial pathogens especially those with zoonotic potential. We examined 276 *Rhipicephalus sanguineus*, 38 *Haemaphysalis elliptica* and 4 *Amblyomma hebraeum* ticks from 90 dogs and 4 cats from the Free State, KwaZulu-Natal, North West and Mpumalanga provinces. DNA of *Coxiella burnetii* (41%), *Ehrlichia or Anaplasma* (18%), *Rickettsia* spp. (37%), *Anaplasma phagocytophilum*-like bacterium (18%) and *Ehrlichia canis* (19%) was detected by polymerase chain reaction (PCR) from a total of 147 pooled DNA samples. All samples were negative for the presence of *Borrelia burgdorferi* DNA. *Ehrlichia canis* was detected in samples from all the provinces except the North West; *A. phagocytophilum* was absent in KwaZulu-Natal samples, whereas *Rickettsia* species and *C. burnetii* were detected in all sampled provinces. The PCR-positive samples were confirmed by direct sequencing of the product. Data from this study calls for a joint effort by both veterinary and medical sectors to conduct epidemiological studies of the zoonotic pathogens in both animals and humans.

## Introduction

In South Africa, a number of studies have been done on tick-borne diseases of canines targeting *Ehrlichia canis, Babesia vogeli* and *Babesia rossi* in host animals (Allsopp & McBride 2009; Matjila et al. 2008; McBride et al. 1996). However, full description of zoonotic pathogens such as *Rickettsia* species, *Borrelia burgdorferi, Anaplasma phagocytophilum* and *Coxiella burnetii* as well as their relationship to emerging and characterised illnesses, is not widely available. The widespread occurrence of tick hosts in South Africa may promote the occurrence of diseases caused by the above-mentioned pathogens as speculated by Fivaz and Petney (1989).

Ehrlichioses are very diverse, but the organisms causing infections in the country have yet to be isolated and characterised. Serological studies have shown that up to 75% of dogs have significant antibody titres against *E. canis* and *Ehrlichia chaffeensis* in Bloemfontein, South Africa (Pretorius & Kelly 1998). DNA of *E. canis* and that of a novel *Ehrlichia* species closely related to *Ehrlichia ruminantium* have been found in the blood of dogs in South Africa. These animals showed clinical signs suggestive of ehrlichiosis, but it could not be confirmed whether the *E. ruminantium-*like organism was the cause of the illness (Allsopp & Allsopp 2001; Inokuma et al. 2005; McBride et al. 1996).

*Anaplasma phagocytophilum* and *B. burgdorferi sensu lato*, the causative agents of human granulocytic anaplasmosis (HGA) and Lyme disease (LD), respectively, are common in North America and Europe (Liebisch, Sohns & Bautsch 1998; Maudlin, Eisler & Welburn 2009). Anecdotal cases of LD (Strijdom & Berk 1996) and a novel *Anaplasma* species closely related to *A. phagocytophilum* detected in canine blood in South Africa (Inokuma et al. 2005) have been described. Despite reports of these pathogens, their true incidence has not been properly investigated.

Rickettsioses caused by *Rickettsia conorii* (Mediterranean spotted fever [MSF]) and *Rickettsia africae* (African tick-bite fever [ATBF]) are the most common forms in sub-Saharan Africa. Although not commonly reported among indigenous people because they do not display clinical signs of the diseases (Kelly 2006; Ndip et al. 2004; Rutherford et al. 2004), they have proven to be problematic to South Africa’s tourism industry, where numerous reports of infection and illness have been reported from tourists returning to their home countries after visiting nature reserves in South Africa (Portillo et al. 2007; Raoult et al. 2001; Roch et al. 2008).

Query fever (Q-fever) caused by *C. burnetii* is distributed across the world, and infected pets in addition to livestock are a potential source of infection to humans (Cooper et al. 2011; Matthewman et al. 1997; Mediannikov et al. 2010). It is transmitted by an array of tick species carrying about 40% natural infection and shedding a significant number of viable organisms in their faeces. *Coxiella burnetii* may also be transmitted via aerosol inhalation (Berri, Laroucau & Rodolakis 2000; Kelly et al. 1993; Mediannikov et al. 2010; Psaroulaki et al. 2006).

As dogs and cats are increasingly spending more time in homesteads in southern Africa, this study sought to determine the occurrence of tick-borne bacterial and rickettsial pathogens especially those with zoonotic potential, such as *R. africae, B. burgdorferi, A. phagocytophilum* and *C. burnetii.* Furthermore, using molecular techniques, we also investigated the occurrence of commonly reported *E. canis* from ticks infesting dogs and cats.

## Materials and methods

### Sampled areas

Ticks were mainly collected from KwaZulu-Natal (KZN), Free State (FS) and North West (NW) provinces of South Africa. Sampling sites included Wesselsneck (S 28° 20′ 0.52″ E 030° 02′ 49.1″), Gcinalishona or Mjindini (adjacent villages) (S 28° 39′ 00.5″ E 030° 06′ 56.3″), Tholeni (S 28° 25′ 39.9″ E 30°13′ 04.3″) and Msinga (S 28° 41′ 43.1″ E 030° 16′ 14.6″) in KZN; Sekoto farm (S 28° 36.094′ E 028° 49.013′) in the FS; and in NW, samples were collected at a private veterinary clinic in Mafikeng (S 25° 51′ 0″ E 25° 38′ 0″). A few samples were also obtained from Kameelpoort-KwaMhlanga (S 25° 46′ 6.3″ E 29° 28′ 42″) in Mpumalanga (MP). In addition to these samples, 45 tick DNA samples were sourced from a previous study conducted by Leodi et al. (2011). The ticks in that study were collected from dogs in Phuthaditjhaba in the FS with no record of tick species identification.

### Tick collection and processing

Ticks were collected from the head (mainly in the ears) and along the whole body of the dogs and cats using fine-tipped forceps. They were transferred into collection vials and later identified to species level using taxonomic keys (Norval & Horak 2004; Walker et al. 2003). Representatives of each identified species were confirmed by a tick taxonomist at the Onderstepoort Veterinary Institute. Fully engorged females were kept at room temperature until they had completed oviposition, whereas unengorged females and males were stored in collection vials containing 75% ethanol. Ticks from each dog and cat were grouped according to their species; one to three ticks of the same species were pooled depending on the number of similar species collected and identified from individual animals. All ticks were surface sterilised twice with 75% ethanol then washed in sterile phosphate-buffered saline (PBS) solution before they were crushed in sterile 1.5 mL Eppendorf tubes (Hamburg, Germany) containing 200 µL of PBS. Egg masses laid in collection vials were washed in PBS, centrifuged at full speed and crushed with a sterile glass rod. Thereafter, DNA was extracted and subjected to polymerase chain reaction (PCR) together with the adult tick samples.

### DNA extraction and polymerase chain reaction

DNA from ticks and egg masses was extracted using the salting out method (Miller, Dykes & Polesky 1998). Pellets were dissolved in 50 µL – 200 µL of double distilled water (depending on the size of the pellet) and stored at -34 °C for further use. The extracted DNA together with the 45 DNA samples from Phuthaditjhaba were subjected to PCR amplification using oligonucleotide sequences listed in Table 1. The samples were screened for the presence of *A. phagocytophilum, B. burgdorferi s. l., C. burnetii, Ehrlichia* or *Anaplasma, E. canis* and *Rickettsia* species. PCR was performed using AmpliTaq Gold^®^ 360 Master Mix (Applied Biosytems, USA) as per manufacturer’s instructions using a Veriti^®^ thermal cycler (Applied Biosystems, USA). Positive controls were obtained from the Research Center for Zoonosis Control (CZC), Hokkaido University, Japan, and from the School of Medicine, Johns Hopkins University.

**TABLE 1:** Oligonucleotide sequences used for polymerase chain reaction amplification of the target pathogens.

Pathogen	Primer sequences	Product size (bp)	References
*Anaplasma phagocytophilum*	EHR-521 – TGT AGG CGG TTC GGT AAG TTA AAG EHR-747 – GCA CTC ATC GTT TAC AGG GTG	250	Welc-Faleciak et al. (2009)
*Borrelia burgdorferi sensu lato*	FL6 – TTC AGG GTC TCA AGC TTG CAC T FL7 – GCA TTT TCA ATT TTA GCA AGT GAT G	276	Picken et al. (1996); Welc-Faleciak et al. (2009)
*Borrelia burgdorferi*	B1 – ATG CAC ACT TGG TGT TAA CTA B2 – GAC TTA TCA CCG GCA GTC TTA	126	Morozova et al. (2002)
*Coxiella burnetii*	CB-1: 59 – ACT CAA CGC ACT GGA ACC GC CB-2: 59 – TAG CTG AAG CCA ATT CGC C	257	Parola and Raoult (2001)
*Ehrlichia canis*	E.c 16S fwd – TCGCTATTAGATGAGCCTA CGT E.c 16S rev – GAGTCTGGACCGTATCTCAGT	154	Peleg et al. (2010)
*Ehrlichia/Anaplasma* spp.	Ehr- F – GGA ATT CAG AGT TGG ATC MTG GYT CAG Ehr-R biotin – CGG GAT CCC GAG TTT GCC GGG ACT TYT TCT	352–460	Matjila et al. (2008)
*Rickettsia* spp.	RpCS-877p – GGGGACCTGCTCACGGCGG RpCS 1273r – CATAACCAGTGTAAA	380	Inokuma et al. (2008)

### Sequencing and data analysis

The positive PCR products were purified using USB ExoSAP-IT Enzymatic PCR Product Clean-Up (Affymetrix Japan K. K., Tokyo, Japan). The forward and reverse primer pairs in Table 1 were utilised in direct sequencing of the purified PCR products. Cycle sequencing reactions were performed using an ABI Prism BigDye Terminator Cycle Sequencing Kit (Applied Biosystems Thermo Fisher Scientific) in an ABI 3130 DNA Sequencer. The sequence data of the PCR products were analysed using BLASTn (National Center for Biotechnology Information, Bethesda, MA, USA; http://www.ncbi.nlm.nih.gov/blast/) for homology searching. Pearson’s χ^2^ test was used to estimate the significant differences between the incidences of each pathogen within the different provinces. MP was excluded from the calculations because of small sample size.

## Results

A total of 318 individual ticks were identified and processed for PCR screening. They were collected from 90 dogs (FS [*n* = 10], KZN [*n* = 63], NW [*n* = 16] and MP [*n* = 1]) and 4 cats from MP. The identified tick species included *Rhipicephalus sanguineus* (*n* = 276), *Haemaphysalis elliptica* (*n* = 38) and *Amblyomma hebraeum* (*n* = 4). From the 318 ticks, 147 pools were made from which DNA was extracted. This DNA together with the DNA extracted from the egg masses (*n* = 17) and the Phuthaditjhaba samples (*n* = 45) were subjected to PCR amplification to detect the target pathogens.

Overall, 18% *Ehrlichia* or *Anaplasma* DNA was detected from the pools among the three provinces (FS, MP and KZN), whereas all NW samples were negative. All the samples were subsequently screened for *E. canis* and *A. phagocytophilum,* respectively. An overall infection rate of 19% for *E. canis* was obtained, with a 100% maximum identity to published *E. canis* sequences upon database analysis (Accession no: CP000107.1, DQ228514.1 & HQ844983.1). Table 2 shows the results obtained in each province. The majority (60%) of the positive samples were from the unidentified specimens, whereas 40% of the positive samples were detected solely from *Rh. sanguineus* species of the identified ticks. The *H. elliptica* and *A. hebraeum* ticks were negative for the presence of *E. canis* DNA.

**TABLE 2:** Overall infection rates of ticks with target pathogens per province.

Provinces	*Coxiella burnetii*		*Rickettsia* sp.		*Ehrlichia*/*Anaplasma*		*Ehrlichia canis*		*Anaplasma phagocytophilum*	
				
	*n*	%	*n*	%	*n*	%	*n*	%	*n*	%
**Dogs**										
FS	30/55	55	28/54	52	9/50	18	13/50	26	11/52	21
KZN	18/56	32	11/52	21	12/55	22	10/55	18	0/55	0
NW	5/16	31	6/16	38	0/16	0	0/16	0	10/16	63
MP	1/1	100	0/1	0	1/1	100	0/1	0	1/1	100
Total	54/128	41	45/123	37	22/122	18	23/122	19	22/124	18
**Cats**										
MP	0/4	0	0/4	0	0/4	0	0/4	0	4/4	100
**Egg masses**										
KZN	3/17	18	4/17	24	2/17	12	2/17	12	0/17	0

Represented as percentage of infection by target pathogen per tick pool in the different provinces.

KZN, KwaZulu-Natal; FS, Free State; NW, North West; MP, Mpumalanga.

**FIGURE 1:**
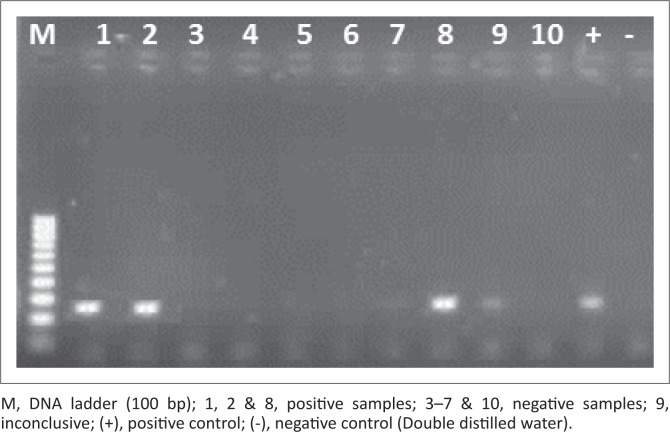
Gel electrophoresis of *Anaplasma phagocytophilum* polymerase chain reaction. Amplified polymerase chain reaction product with amplicon size of 250 bp.

For *A. phagocytophilum*, 18% of the total samples tested positive and gave a corresponding band of 250 bp during electrophoresis (Figure 1). Only 15% of the *A. phagocytophilum* PCR positive samples had been positive for the presence of *Ehrlichia/Anaplasma* DNA. In NW, the infection rate with *A. phagocytophilum* was as high as 63% (Table 2). We directly sequenced portions of the 16S rRNA gene amplified from the ticks that were positive for the bacterium by PCR. The sequences matched with uncultured *Anaplasma* species (KX417200; KX417196 and KX417195) with identity ranging from 91% to 93% and coverage ranging from 62% to 88%. Both *Rh. sanguineus* (50%) and *H. elliptica* (15%) species carried the pathogen but it was not detected in *Amblyomma* ticks. The other 35% of the positive samples can be attributed to the unidentified tick species samples.

In addition to amplification of *A. phagocytophilum* and *E. canis*, sequence analysis of *Ehrlichia*/*Anaplasma* positive samples pointed towards the presence of an *Anaplasma* species with 96% similarity to *Anaplasma marginale* (Accession no. LC126880).

**FIGURE 2:**
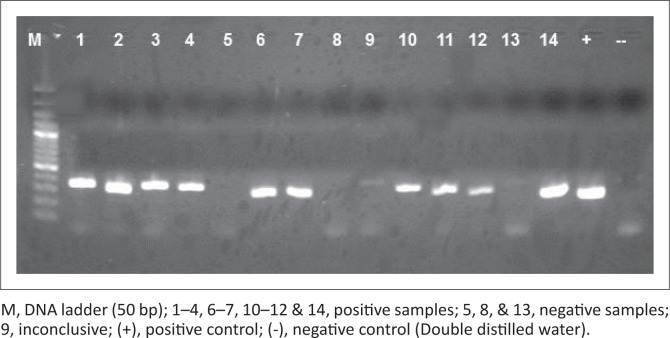
Gel electrophoresis of *Coxiella burnetii* polymerase chain reaction. Amplified polymerase chain reaction product with amplicon size of 257 bp.

*Coxiella burnetii* infection rate was 41% among all the provinces. Infection rates per province ranged between 31% and 55% (Table 2). Sequence analysis of *C. burnetii* PCR-positive samples (Figure 2) revealed 99% maximum identity with *C. burnetii* CbUKQ154 complete genome (GenBank accession no: CP001020) and *C. burnetii* R.S.A.331 complete genome (GenBank accession no: EU448153.1). Of the total positive samples, the highest infection was 52%, obtained among the unidentified dog-tick samples, followed by 44% *Rh. sanguineus* and lastly, 4% in *H. elliptica.* Of the four *Amblyomma* ticks, only one showed the presence of *C. burnetii* DNA.

**FIGURE 3:**
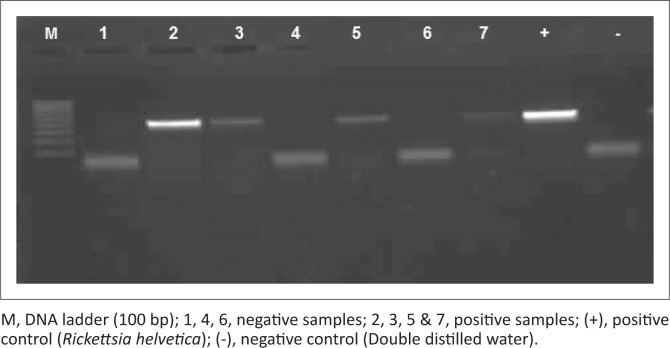
Gel electrophoresis of *Rickettsia* species polymerase chain reaction. Amplified polymerase chain reaction product with amplicon size of 401 bp.

*Rickettsia* species were detected in the FS, KZN and NW, but were absent in samples from MP, giving an overall 37% prevalence of infection. To determine the actual *Rickettsia* species amplified (Figure 3), the gltA gene was sequenced revealing a 99% – 100% identity with *R. africae* glt gene partial sequences for all of the samples and 5% of these were also 99% identical to *R. conorii* strains (Accession no: U59728.1, U59733.1 & AE006914.1). Sixty-five per cent of the positive samples were among the unidentified tick species, whereas 35% were detected in *Rh. sanguineus.* The *A. hebraeum* and *H. elliptica* pools were negative for the presence of *Rickettsia* DNA.

*Borrelia burgdorferi s. l.* could not be detected from any of the tick samples from dogs and cats using the two primer sets in Table 1. Tick samples obtained from cats were all PCR positive for the *A. phagocytophilum*-like bacterium and none of the other target pathogens.

Only the KZN ticks laid eggs within the collection vials from which the 17 DNA samples were obtained. The ticks that laid eggs were all identified as *Rh. sanguineus.* All the samples were negative for *A. phagocytophilum* and *B. burgdorferi s. l*., but 18% *C. burnetii*, 12% *E. canis* and 24% *Rickettsia* species DNAs were detected (Table 2). Distribution of the target pathogens varied quite significantly according to sampled area (χ^2^ = 95.679, *df* = 6, *p* < 0.05). Co-infections with two pathogens were observed among 24% of *Rh. sanguineus* and 24% of *H. elliptica* ticks, whereas 7% co-infections with three pathogens were observed overall for the identified tick species.

## Discussion

*Ehrlichia* or *Anaplasma* PCR primers were used as a preliminary form of screening for the presence of *E. canis* and *A. phagocytophilum*. As can be seen from the results, the primers were more biased towards *E. canis* than *A. phagocytophilum*, and therefore, species-specific primers were then utilised. Subsequently, the target pathogens were amplified.

In this study, the species-specific PCR assay was positive for the *A. phagocytophilum*-like infections from ticks collected from dogs. An *A. phagocytophilum-*like organism was previously reported from whole blood samples of three dogs in Bloemfontein, identified and characterised by microscopic observation and PCR but was found to (although closely related) differ significantly from *A. phagocytophilum* (Inokuma et al. 2005; Matjila et al. 2008). Although we reported the presence of *A. phagocytophilum* in ruminants (Mtshali et al. 2015), the positive detection of this pathogen from dogs, however, requires further genetic characterisation studies, as sequences obtained from this study matched with uncultured *Anaplasma* species reported from blood of sheep.

From our results, it is clear that the *A. phagocytophilum-*like bacterium exists in ticks infesting dogs in South Africa, but is probably maintained within the cycle between dogs (Inokuma et al. 2005; Matjila et al. 2008), non-canine intermediate hosts of the tick and the ticks themselves. Concerning transmission to humans, the *Ixodes persulcatus* group of ticks (vectors of HGA) is absent in the country, which could explain the lack of disease reports. In the absence of *I. perculcatus, Rh. sanguineus* and *H. elliptica* should be considered as possible vectors. The actual incidences of this bacterium, as well as their true hosts in South Africa, remain unknown.

The 19% rate of infection with *E. canis* observed was detected solely from *Rh. sanguineus* ticks. None of the identified *H. elliptica* or *A. hebraeum* ticks carried *E. canis.* This is probably because of its high affinity for *Rh. sanguineus* as a carrier (Harrus & Waner 2005). Its absence from the NW ticks could possibly be attributed to frequent use of doxycyclines in well cared for dogs sampled in this study that are presented, examined and treated at the veterinary clinic (unpublished observations).

*Coxiella burnetii* is found all over the African continent, with generally higher serological indices reported for *Amblyomma variegatum, Hyalomma truncatum* and *Rhipicephalus senegalensis* ticks in several West African countries (Mediannikov et al. 2010). Its incidence, however, is unknown and may be underestimated (Fournier, Marrie & Raoult 1998). In South Africa, *C. burnetii* infections in canines have only been demonstrated in wild dogs (*Lycaon pictus*) in the Kruger National Park by serological methods (Van Heerden et al. 1995), whereas a 2% seroprevalence has been reported in feline blood (Frean & Blumberg 2007). We could not find reports of tick infections with *C. burnetii* in South Africa prior to our previously published study (Mtshali et al. 2015), where we positively identified the pathogen in KZN and the FS from ticks collected from ruminants (sheep, goats and cattle) with infection rates ranging between 20% and 68% among the sampled groups. In a similar study, Halajian et al. (2016) could not detect *C. burnetii* infections in sheep, cattle and wildlife in Limpopo and Western Cape provinces. Nevertheless, in this study we report a notably high prevalence of 41% when compared with 7.8% found in a similar study in Cyprus (Psaroulaki et al. 2006) as well as to the above-mentioned studies. This infection rate is worrisome as in some regions, pets are actually more commonly implicated in the transmission of Q-fever to humans (Cooper et al. 2011). Humans can potentially be infected through various ways inclusive of tick bites (Porter et al. 2011). The prevalence of *C. burnetii* in humans is unknown in South Africa, despite the fact that it was described as the most prevalent rickettsial infection prior to its reclassification (Frean & Blumberg 2007).

The present infection rates of *R. africae* and *R. conorii*, which may be as high as 52%, are a cause for concern. In this investigation, the rickettsiae were detected exclusively from *Rh. sanguineus* ticks with low infection rates. This is a tick species with a relatively low affinity for humans and not known to biologically transmit *R. africae* (Fournier et al. 1998). On the contrary, *A. hebraeum* has the potential (75%) for transmitting rickettsiae of medical importance (Fournier, Beytout, J. & Raoult 1999; Prabhu et al. 2011), but the ticks tested negative for the pathogen.

It is, however, difficult to quantify the risk factor, because the role of dogs and cats as reservoirs of infection is undetermined. This is because of the fact that animals with rickettsial infections remain asymptomatic and are bacteraemic for short periods only (Vorou, Papavassiliou & Tsiodras 2007). On the contrary, Frean and Blumberg (2007) state that dogs, rodents and ticks are sources of human infection that transmit *R. conorii* (via *Rh. sanguineus*) in peri-urban areas and *R. africae* (via *A. hebraeum*) in rural areas. Seroconversion in indigenous residents results from inconspicuous infections whereas infection of tourists may manifest as MSF and Boutonese-fever like TBF.

From our results, we could deduce that *C. burnetii, E. canis* and *Rickettsia* species are transovarially transmitted as they were positively detected by PCR from DNA extracted from eggs, although no comparisons were made between the eggs and the adult females. Nevertheless, according to the literature, *E. canis* is said to be transmitted only transstadially and not transovarially, whereas *Rickettsia* is transmitted both transstadially and transovarially (Taylor, Coop & Wall 2007). In the case of *C. burnetii*, it is said to be transovarially transmitted but it is also possible that the eggs may become positive because of contamination from coxal and faecal secretions of positive adult ticks shedding viable organisms as previously reported (Psaroulaki et al. 2006). Regardless of these findings, it is clear that ticks play an important role in maintaining viability of the target pathogens in nature. The results also prove that distribution of pathogens is largely dependent on their affinity to the tick in the case of *Rickettsia* and *Ehrlichia*, whose vectorial capacity has been defined, but the same cannot be said about *A. phagocytophilum* and *C. burnetii.* Tick and pathogen species variation also depended on the sample size per sampled area and lastly, ticks can be infected with multiple pathogens simultaneously, with as many as three recorded in this study.

Generally, laboratory capacity to diagnose these infections in humans is often lacking in developing countries (Kelly et al. 1996; Prabhu et al. 2011). Consequently, the importance of *Coxiella, Rickettsia, Ehrlichia, Anaplasma* and *Borrelia* as causes of illness in sub-Saharan Africa is poorly characterised. The studied pathogens must be considered seriously as they have the capacity to cause human communicable diseases. Further studies are required to shed more light on their epidemiology, through characterisation of species, their distribution in the country, determination of pathogenicity in dogs and cats and perhaps other species as well as determination of the vectorial capacity of the tick species identified in this study and those occurring throughout South Africa. Furthermore, measures to control the ticks and these pathogens would improve animal welfare and contribute to improved public health.

## Conclusion

This study has documented the occurrence of *Rickettsia* species, *Anaplasma* species, *E. canis* and *C. burnetii* in ticks (*A. hebraeum, H. elliptica* and *Rh. sanguineus*) collected from dogs and cats using PCR. Vectorial capacity of these ticks for above mentioned pathogens to dogs and cats needs to be determined in future studies. This study has further detected DNA of *Rickettsia* species, *Anaplasma* species, *E. canis* and *C. burnetii* from eggs of *Rh. sanguineus*, indicating that there is transovarial transmission of these pathogens from infected engorged female ticks to egg stage. This observation calls for further investigation of transtadial transmission of these pathogens during the tick life cycle stage development. Nevertheless, this study has demonstrated that tick-borne zoonotic pathogens of genera *Anaplasma, Erhlichia, Rickettsia* and *Coxiella burnetii* are prevalent in tick vectors collected in dogs from some provinces of South Africa.
